# Dysregulated Estrogen Receptor Signaling in the Hypothalamic-Pituitary-Ovarian Axis Leads to Ovarian Epithelial Tumorigenesis in Mice

**DOI:** 10.1371/journal.pgen.1004230

**Published:** 2014-03-06

**Authors:** Mary J. Laws, Athilakshmi Kannan, Sandeep Pawar, Wanda M. Haschek, Milan K. Bagchi, Indrani C. Bagchi

**Affiliations:** 1 Department of Comparative Biosciences, University of Illinois Urbana/Champaign, Urbana, Illinois, United States of America; 2 Department of Molecular and Integrative Physiology, University of Illinois Urbana/Champaign, Urbana, Illinois, United States of America; 3 Department of Pathobiology, University of Illinois Urbana/Champaign, Urbana, Illinois, United States of America; University of Washington, United States of America

## Abstract

The etiology of ovarian epithelial cancer is poorly understood, mainly due to the lack of an appropriate experimental model for studying the onset and progression of this disease. We have created a mutant mouse model in which aberrant estrogen receptor alpha (ERα) signaling in the hypothalamic-pituitary-ovarian axis leads to ovarian epithelial tumorigenesis. In these mice, termed ERα^d/d^, the ERα gene was conditionally deleted in the anterior pituitary, but remained intact in the hypothalamus and the ovary. The loss of negative-feedback regulation by estrogen (E) at the level of the pituitary led to increased production of luteinizing hormone (LH) by this tissue. Hyperstimulation of the ovarian cells by LH resulted in elevated steroidogenesis, producing high circulating levels of steroid hormones, including E. The ERα^d/d^ mice exhibited formation of palpable ovarian epithelial tumors starting at 5 months of age with 100% penetrance. By 15 months of age, 80% of ERα^d/d^ mice die. Besides proliferating epithelial cells, these tumors also contained an expanded population of luteinized stromal cells, which acquire the ability to express P450 aromatase and synthesize E locally. In response to the elevated levels of E, the ERα signaling was accentuated in the ovarian epithelial cells of ERα^d/d^ mice, triggering increased ERα-dependent gene expression, abnormal cell proliferation, and tumorigenesis. Consistent with these findings, treatment of ERα^d/d^ mice with letrozole, an aromatase inhibitor, markedly reduced circulating E and ovarian tumor volume. We have, therefore, developed a unique animal model, which serves as a useful tool for exploring the involvement of E-dependent signaling pathways in ovarian epithelial tumorigenesis.

## Author Summary

Ovarian cancer is currently the most lethal gynecological cancer in the United States. Multiple epidemiological studies indicate that women who take hormone replacement therapy, estrogen or estrogen with progesterone, peri- or postmenopause will have an increased chance of developing ovarian cancer. Unfortunately, the five-year survival rate after diagnosis is very low indicating that better tools are needed to diagnose and treat ovarian cancer. The models that would allow investigation of this disease are severely limited. In this article we introduce a mouse model that develops epithelial ovarian tumors, and by employing inhibitors of estrogen synthesis, we show that ovarian tumorigenesis in this model is dependent on estrogen production within the ovarian tumor. These studies suggest that estrogen may play a role in promoting ovarian tumor growth.

## Introduction

Ovarian cancer is the most lethal malignancy of the female reproductive system and the fifth leading cause of cancer-related death among women [1]. Approximately 90% of malignant ovarian tumors are derived from either the ovarian surface epithelium (OSE) or fallopian tube epithelium (FTE) [2]. Due to the absence of specific symptoms and the lack of strategies for early detection of ovarian cancer, the majority (70%) of women with this disease are diagnosed at a late stage when the cancer has spread beyond the confines of the ovary [1]. Despite its clinical significance, the etiology of ovarian cancer is poorly understood, mainly due to the lack of an appropriate experimental model for studying the onset and progression of this disease.

Multiple theories regarding the etiology of ovarian cancer have been proposed, but the precise molecular defects underlying the development of this disease remain elusive [3]. The “gonadotropin hypothesis” proposes that high gonadotropin levels can have a stimulatory effect on OSE cells, promoting their neoplastic transformation [4], [5]. It was reported that the addition of gonadotropins to rodents in which ovarian cancer was induced upon treatment with the chemical carcinogen, 7,12-dimethylbenz(a)anthracene (DMBA) led to increased lesion severity, suggesting that gonadotropins play a role in tumor progression [6]. In humans, epidemiologic evidence, indirectly supporting this hypothesis, includes the well-documented protective effects of oral contraceptives and multiparity, which suppress gonadotropin secretion by the pituitary gland [5], [7]. The majority of women with epithelial ovarian cancer present the disease at a postmenopausal stage where circulating follicle stimulating hormone (FSH) and lutenizing hormone (LH) levels are elevated, indicating a causal relationship between chronically elevated gonadotropin levels and ovarian cancer development [5], [8].

Besides gonadotropins, epidemiological studies have reported altered ovarian cancer risk associated with the use of steroid hormones to ease menopausal symptoms. Estrogen (E) is a well-known mitogenic factor associated with the genesis of many cancers. It has been reported previously that the risk of developing ovarian cancer increases in women who use hormone replacement therapy (HRT) for more than five years or use E-only regimens [9]–[13]. While most of these studies comprise a small number of subjects and fail to control for all of the factors that may influence cancer risk, in patients with ovarian cancers, intratumoral production of E via *in situ* aromatization has been suggested to promote growth of breast, endometrial and ovarian cancer cells [14]. However, only few animal models have been used to investigate the role of E in ovarian tumorigenesis. Bai *et al* reported the effects of prolonged E exposure on the morphology of rabbit ovaries and found an increase in both OSE cell proliferation and the number of papillae covering the ovarian surface, but no ovarian tumors [15]. In a recent study, Laviotte *et al* conditionally activated an oncogene, SV40 TAg, in OSE cells and treated the mice with exogenous E [16]. These investigators reported that E treatment resulted in an earlier onset of ovarian tumors and a significantly decreased survival time [16]. While the results from this animal model underscore the importance of E in the progression of ovarian cancer, it is clear that new animal models independent of specifically directed single oncogenic mutations are needed for assessment of the role of E signaling in ovarian epithelial tumorigenesis.

In this study, we present a novel transgenic mouse model of ovarian tumorigenesis. In this model, termed ERα^d/d^, the estrogen receptor alpha (ERα) gene is dysregulated in the hypothalamic-pituitary-ovarian axis. Conditional deletion of this gene in the anterior pituitary, but not in the hypothalamus and the ovary, led to elevated circulating LH. Hyperstimulation by LH resulted in luteinization of the ovarian stromal cells, expression of P450 aromatase in these cells, and increased E synthesis in the ovarian microenvironment. Our study suggests that E critically controls ovarian tumor growth, presumably by stimulating the proliferation of OSE cells to drive epithelial tumorigenesis. The ERα^d/d^ mouse, therefore, provides a useful model to study the mechanisms by which dysregulated E signaling promotes the initiation and progression of ovarian epithelial tumors.

## Results

**Figure 1:**
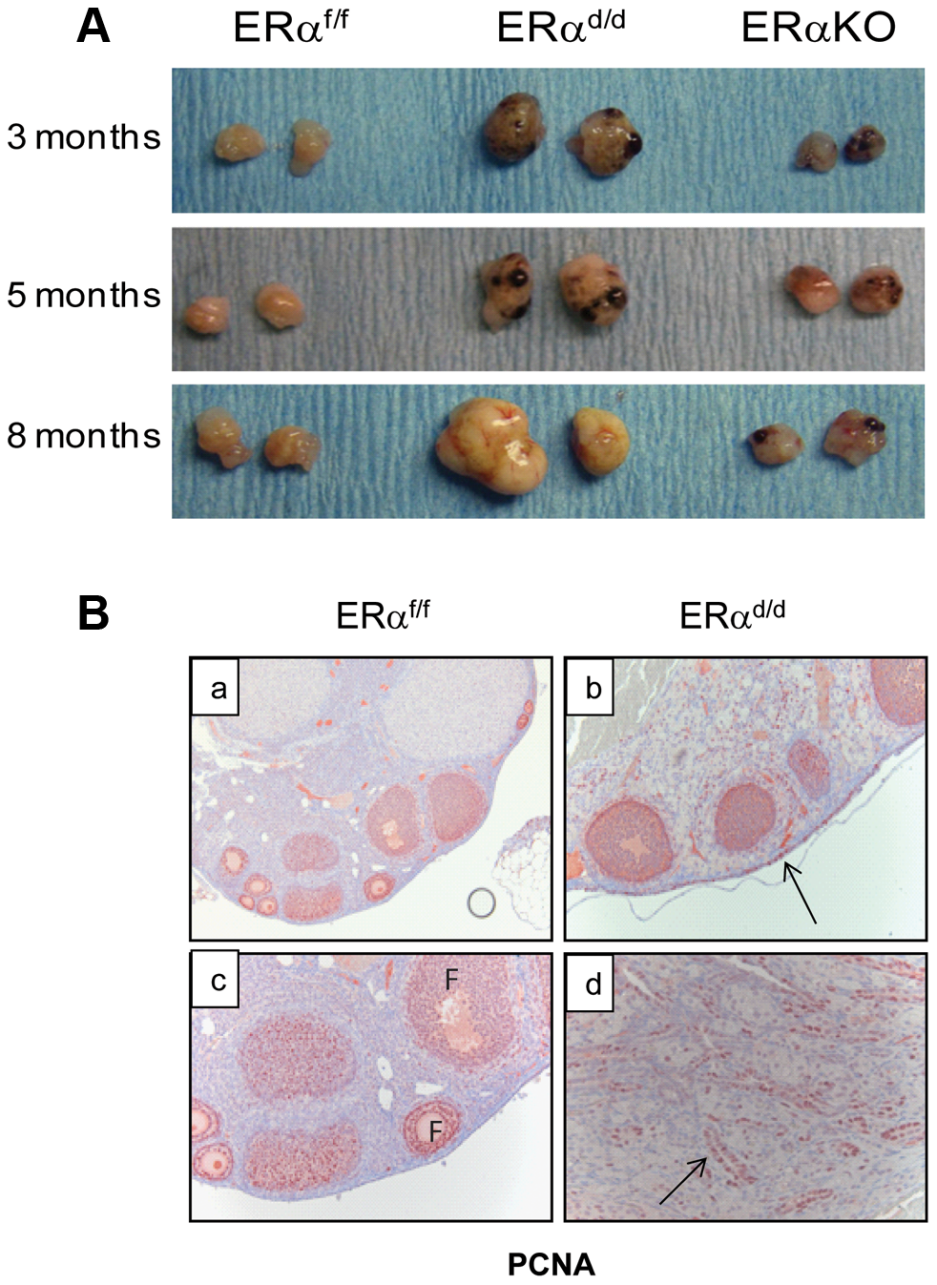
The ERα^d/d^ mice form proliferative ovarian tumors. (**A**) Gross morphology of ERα^d/d^, ERα^f/f^, and ERα global KO mouse ovaries at 3, 5, and 8 months of age. (**B**) Immunohistochemistry of ERα^f/f^ ovary (panels a, c) and ERα^d/d^ ovaries (panels b, d) with tumors at 6 months of age using anti-PCNA antibody. Red staining indicates proliferating PCNA positive cells. Arrows point to hyperproliferative OSE (b) and tumor cells (d) in ERα^d/d^ ovarian tumors. F indicates follicle.

ERα conditional knockout mice (ERα^d/d^) were generated by crossing progesterone receptor cre recombinase (PR-Cre) knock-in mice with ERα floxed (ERα^f/f^) mice [17], [18]. By five months of age, the ERα^d/d^ mice developed palpable ovarian tumors with 100% penetrance. In contrast, the ERα^f/f^ and the global ERα knockout mice did not develop any tumor (Fig. 1A). The ovarian tumors of ERα^d/d^ mice grew progressively with age and became as large as 11 mm in size with an average weight of 300 mg by eight months of age (Fig. S1A). Because of this large tumor burden, 80% of the ERα^d/d^ mice die by 68 weeks of age (Fig. S1C). Histological analyses of the ERα^d/d^ ovaries showed cystic hemorrhagic follicles at 3 months of age. By 6 months, there was evidence of neoplastic epithelial cells migrating into the ovarian stroma, and by 11 months, extensive cellular proliferation occurred, resulting in the formation of a large tumor mass (Fig. S1B). Immunohistochemical analysis of ovaries of ERα^f/f^ mice at 6 months of age, using cell proliferation markers, revealed that the follicular granulosa cells were proliferative but the OSE cells were quiescent (Fig. 1B, panel a,c). In sharp contrast, both OSE and the tumor cells within ERα^d/d^ ovaries exhibited pronounced proliferative activity (panels b, d; proliferative cells indicated by arrow) (Fig. 1B).

**Figure 2:**
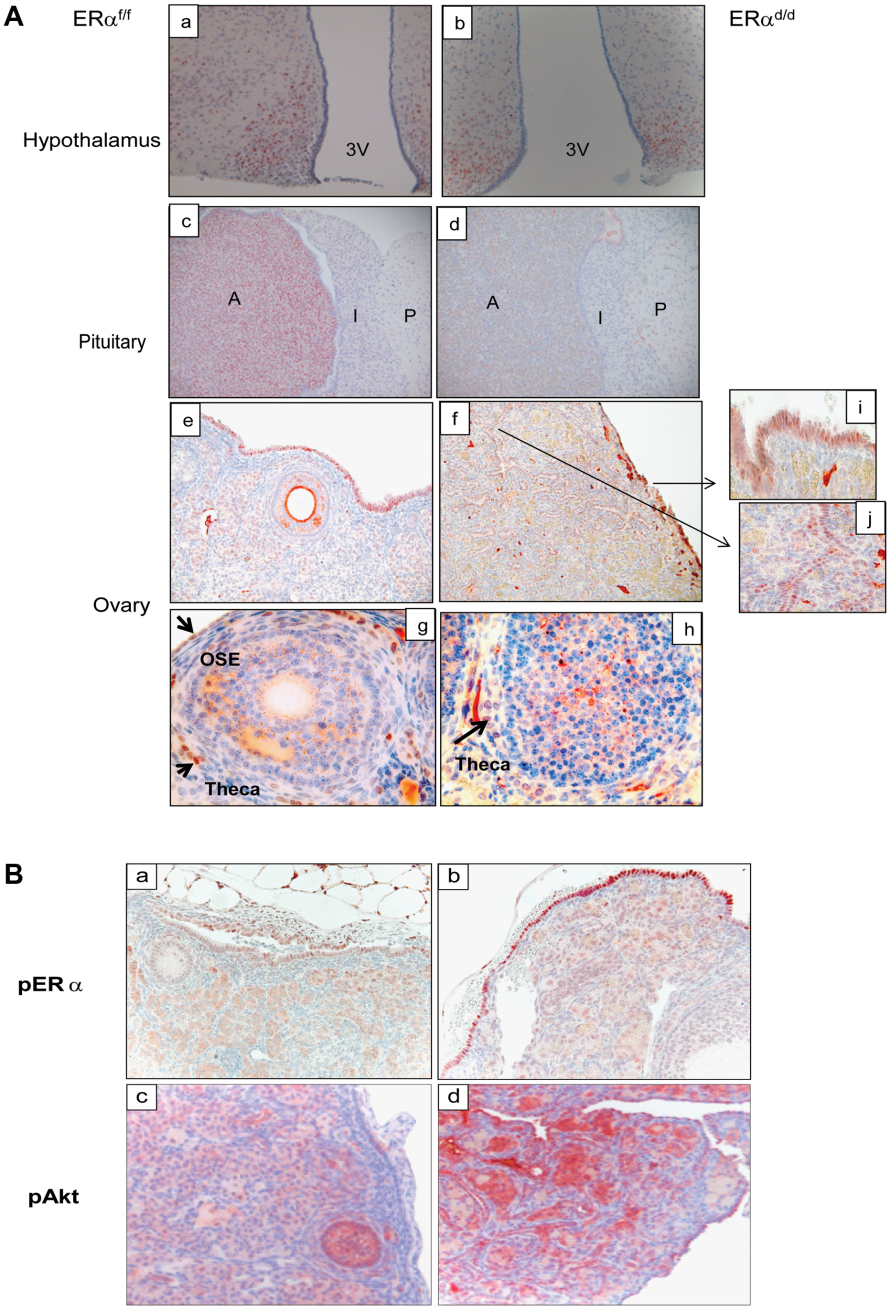
ERα localization in the tissues of HPO axis. (**A**) Histological sections of ERα^f/f^ (a) and ERα^d/d^ (b) hypothalami, ERα^f/f^ (c) and ERα^d/d^ (d) pituitaries, ERα^f/f^ ovary (e, g) and ERα^d/d^ ovarian tumor (f, h) from adult mice at 6 months of age stained with anti-ERα. Inserts i and j indicate higher magnification depicting ERα positive cells (red staining) in the OSC and ovarian tumor cells. 3 V indicates the third ventricle. A indicates the anterior lobe, I indicates the intermediate lobe, and P indicates the posterior lobe of the pituitaries. Arrows point to OSE cells or theca cells expressing ERα. (**B**) Ovarian sections obtained from ERα^f/f^ (left pictures) and ERα^d/d^ (right pictures) mice were subjected to immunohistochemistry using antibodies against phospho-ERα (S118) (panels a, b) and phospho-Akt (S473) (panels c,d).

We next assessed the expression of ERα in the key tissues of the hypothalamic-pituitary-ovarian (HPO) axis. As shown in Fig. 2A, ERα expression was detected near the third ventricle of the hypothalamus in ERα^f/f^ mice, and this expression remained intact in ERα^d/d^ mice. Widespread expression of ERα was also observed in the anterior pituitary of ERα^f/f^ mice. However, the pituitary expression of ERα was absent in ERα^d/d^ mice. The ERα expression was evident in OSE of ERα^f/f^ mice and remained intact in ERα^d/d^ OSE (panels e,f). In addition theca cell expression of ERα also remained intact in the ERα^d/d^ ovaries (panel h). Most notably, ERα was present in the tumor cells of ERα^d/d^ ovaries (inset j, Fig. 2A). The Cre-mediated excision of the floxed ERα gene in the anterior pituitary is consistent with earlier reports indicating high levels of progesterone receptor (PR) expression in this tissue. The lack of Cre-mediated excision of the ERα gene in the hypothalamus and OSE, on the other hand, is presumably due to relatively low levels of PR expression in these tissues [18], [19].

Due to selective ablation of pituitary ERα expression, the ERα^d/d^ mice are likely to experience a loss of negative-feedback regulation by E at the level of pituitary. Consistent with this prediction, the serum level of LH was significantly elevated in ERα^d/d^ mice (Table 1). Hyperstimulation of ovarian cells by LH resulted in increased steroidogenesis, leading to high circulating levels of progesterone, testosterone and E in ERα^d/d^ mice (Table 1). In contrast, the level of FSH was not statistically different between ERα^d/d^ and ERα^f/f^ mice. According to previous reports, the levels of LH, progesterone, testosterone, and E are also elevated in ERαKO mice [19], [20]. Consistent with the ERαKO mouse phenotype, ERα^d/d^ mice are infertile. Adult mice fail to ovulate due to chronic high levels of LH. Due to the lack of ERα expression in uterine epithelial and stromal cells, the ERα^d/d^ uteri are unable to receive an implanting embryo. Furthermore, uterine tumors are not found in the ERα^d/d^ mice, presumably because the major uterine cell types do not express ERα. However, in contrast to the ERαKO mice, which lack ERα in all cells, including the ovarian cells, ERα was intact in OSE of ERα^d/d^ mice. This raised the possibility that elevated systemic E levels contribute to tumor initiation by stimulating ER signaling in OSE of ERα^d/d^ mice but fails to do so in OSE of ERαKO mice.

**Table 1:** Serum hormone measurements of ERα^f/f^ and ERα^d/d^ mice at six months of age.

Hormone	ERα^f/f^	ERα^d/d^	p-value
Estradiol (pg/ml)	7.03+/−.84	63.51+/−36.30	p<.001
Progesterone (ng/ml)	1.94+/−.61	8.78+/−.77	p<.001
LH (ng/ml)	.17+/−.12	2.31+/−.51	p<.001
FSH (ng/ml)	6.30+/−1.58	2.92+/−.57	p = .09
Testosterone (ng/ml)	3.01+/−1.35	394.85+/−59.06	p<.001

Hormone levels from 6 mice were measured for each assay.

In agreement with this view, we observed marked up-regulation of a transcriptionally active form of ERα, phosphorylated at serine 118, in OSE of ERα^d/d^ mice (Fig. 2B, b). We also examined the status of the phosphoinositide 3-kinase (PI3K)/AKT pathway, which is reported to be activated in response to E treatment of ovarian cancer cell lines [21]–[23]. We noted that the level of AKT phosphorylated at Ser 473 (p-AKT) is elevated in the OSE and tumor cells of ERα^d/d^ ovaries, while p-AKT level is maintained at a low level in ERα^f/f^ ovaries (Fig. 2B, c,d). It is likely that the increased level of phosphorylated AKT is linked to the elevated E signaling in ERα^d/d^ ovaries.

**Figure 3:**
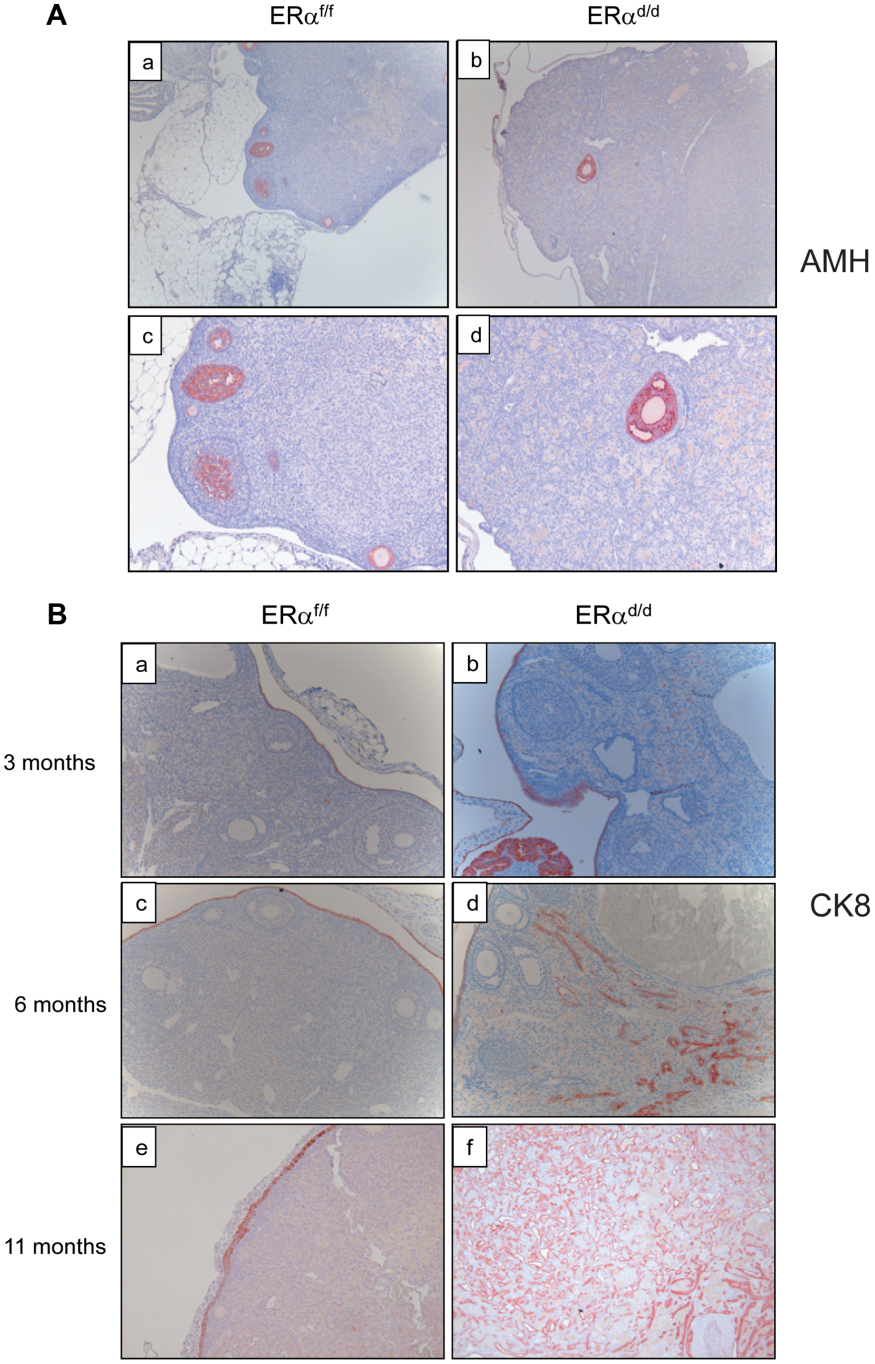
Development of ovarian tumors in ERα^d/d^ mice. (**A**) Ovarian sections from ERα^f/f^ (a, c) and ERα^d/d^ (b, d) mice at 11 months of age were subjected to immunohistochemistry using an antibody against AMH. Red staining indicates AMH positive granulosa cells. (**B**) Ovarian sections from ERα^f/f^ mice at 3, 6, and 11 months of age (left panels a, c, e respectively) and ERα^d/d^ mice at 3, 6, and 11 months of age (right panels b, d, f respectively) were subjected to immunohistochemistry using anti-cytokeratin 8 antibody. Red staining indicates cytokeratin 8 positive epithelial cells.

To further characterize the nature of the ovarian tumor in ERα^d/d^ mice, we performed immunohistochemical analyses using epithelial and granulosa cell biomarkers. Anti-mullerian hormone (AMH) is a well-known marker for normal granulosa cells and granulosa cell tumors [24]. While both ERα^f/f^ and ERα^d/d^ ovaries expressed AMH exclusively in the granulosa cells of follicles, ERα^d/d^ ovaries did not express AMH in the tumor cells, indicating that these tumors are not of granulosa cell origin (Fig. 3A). Analysis using anti-cytokeratin 8 (CK8) antibody revealed that ERα^f/f^ mice express this epithelial marker exclusively in a single layer of OSE at 3, 6, and 11 months of age (Fig. 3B, panels a,c,e). In contrast, the OSE of ERα^d/d^ mice at 3 months of age exhibited multiple layers of cytokeratin-positive cells (panel b). At 6 months of age, we observed pronounced cytokeratin 8 expression within the ovaries of ERα^d/d^ mice, indicating the presence of epithelial cells within the tumor mass (panel d). By 11 months of age, widespread cytokeratin 8 immunostaining was observed within the ovarian tumor, highlighting its remarkable epithelial component (Fig. 3B, panel f).

**Figure 4:**
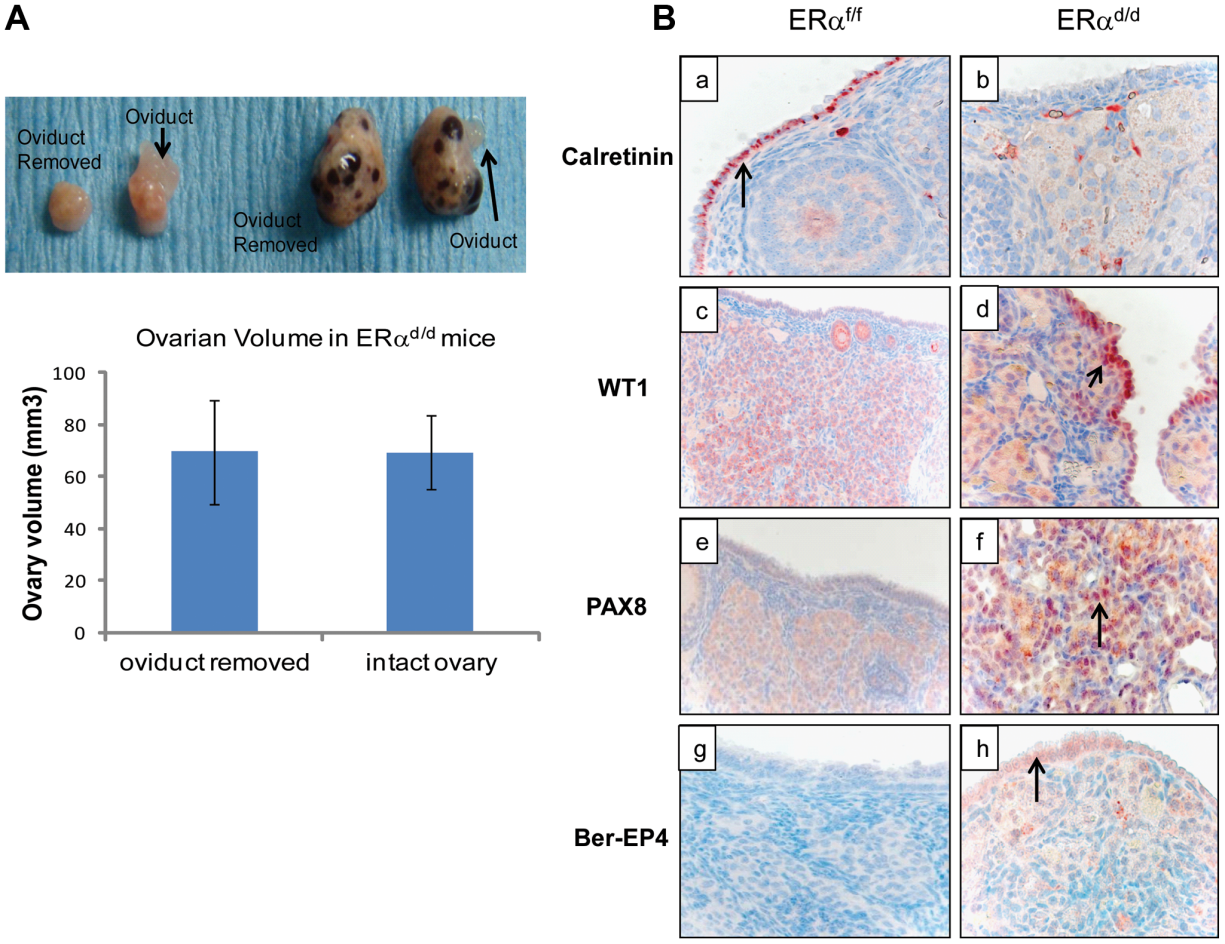
OSE is the site of origin for ovarian tumorigenesis in ERα^d/d^ mice. (**A**) Ovarian tumor growth in ERα^d/d^ mice after surgical removal of oviducts. The right oviduct was surgically removed from ERα^f/f^ and ERα^d/d^ mice at four weeks of age, leaving the left oviduct intact as an internal control. **Upper panel**, Gross morphology of ERα^f/f^ and ERα^d/d^ ovaries at 5 months after oviduct removal surgery. **Lower panel**, The graph depicts ovarian volume comparing ERα^d/d^ tumors with and without the oviduct. Two-tailed t-test was performed for statistical analysis: n = 5; p = 0.99. (**B**) Immunohistochemistry of ERα^f/f^ (left panel) and ERα^d/d^ (right panel) ovaries from mice at 6 months of age using calretinin (a, b); WT1 (c, d); PAX8 (e, f); and Ber-EP4 (g, h) antibodies. Red staining indicates immunostaining.

Current literature suggests that the human ovarian epithelial tumors are derived from either OSE or FTE [2], [25]. Although these epithelia are derived from a common embryologic precursor, OSE is thought to retain mesothelial characteristics, while FTE is terminally differentiated [25]–[27]. Recent studies on the serous subtype of ovarian cancer have suggested that either OSE differentiates to resemble FTE or the cancer originates in the fallopian tube and spreads to the ovary [27]. To investigate further the origin of epithelial ovarian tumor cells in ERα^d/d^ mice, we removed the oviducts of these mice prior to tumor formation. Interestingly, removal of the oviducts from pre-pubertal ERα^d/d^ mice did not prevent the onset of ovarian tumor growth in these animals, indicating that the tumor cells originate from the OSE rather than the oviductal epithelium (Fig. 4A). We also examined the epithelia of ERα^f/f^ and ERα^d/d^ ovaries by monitoring the expression of biomarkers specific for either OSE or FTE. As shown in Fig. 4B, we detected prominent expression of calretinin, a mesothelial marker [2], [28], in OSE of ERα^f/f^ ovaries but not in OSE of ERα^d/d^ ovaries. We also noted marked up regulation of tubal-specific makers, including PAX8, WT1, and Ber-EP4 in the ovaries of ERα^d/d^ mice, while the ovaries of ERα^f/f^ mice lacked their expression. Since PAX8, WT1, and Ber-EP4 are normally expressed in FTE and are present in serous epithelial ovarian tumors [2], [28]–[30], it is likely that the OSE cells of ERα^d/d^ ovaries have undergone differentiation to resemble FTE. Furthermore, it has been reported previously that PAX8 is expressed in serous, endometrioid, and mucinous ovarian cancer while expression of WT1 is restricted to the serous subtype of ovarian cancer [29]. Currently there are no available biomarkers that can differentiate between high and low-grade serous ovarian carcinoma. It is clear that the ERα^d/d^ tumors do not grow aggressively.

To investigate the molecular pathways underlying ovarian tumorigenesis in ERα^d/d^ mice, we next performed gene expression profiling, using RNA isolated from the ovaries of ERα^f/f^ and ERα^d/d^ mice. We identified more than 2500 genes that were differentially expressed in the tumor tissue compared to the normal ovaries. The GEO accession number for the microarray data is GSE39402. When we compared the differentially regulated genes to three different datasets of differential gene expression profiles of human serous adenocarcinoma versus control human ovaries that exist in the Oncomine database, we noted that a large number of genes, which are differentially expressed in human serous ovarian cancer specimens, are also present in ERα^d/d^ ovarian tumors (Fig. S2A). Remarkably, the identity of genes expressed in ERα^d/d^ ovaries and human serous ovarian cancer ranged from 25–40%. Prominent among these genes were those encoding platelet derived growth factor receptor alpha (PDGFRα), vascular cell adhesion molecule (VCAM), clusterin, intercellular adhesion molecule 1 (ICAM-1), and serine/threonine phosphatase 1 (Wip1), which are overexpressed in human serous ovarian cancer [31]–[36]. We observed that the levels of PDGFRα, VCAM, ICAM1, and clusterin were markedly elevated in the ovaries of ERα^d/d^ mice compared to those of ERα^f/f^ mice (Fig. S2B). Collectively, the presence of these cancer biomarkers in ERα^d/d^ ovarian tumors underscored the importance of this model in deciphering the pathways involved in genesis and progression of epithelial ovarian tumorigenesis.

Although the elevated systemic levels of E in ERα^d/d^ mice likely contribute to the initiation of ovarian tumors by stimulating ERα signaling in OSE, we considered the possibility that, as the follicles are depleted with tumor progression, intratumoral E biosynthesis becomes a major regulator of tumorigenesis. Studies in postmenopausal women reported significantly increased expression and activity of P450 aromatase in serous ovarian carcinomas, but not in benign adenomas, supporting the view that intratumoral E derived from *in situ* aromatization could function as an autocrine growth regulator for cancer cells [14], [37]. Previous studies have also reported elevated aromatase activity in tumors and ovarian cancer cell lines [38]–[41]. We observed that ovarian tumors of ERα^d/d^ mice do indeed express high levels of P450 aromatase mRNA (Fig. S3A). To localize aromatase expression we digested ERα^d/d^ ovarian tumors into single-cell suspension, plated both fibroblast stromal and epithelial cells, and completed immunocytochemistry co-localizing both aromatase and a marker indicating the cell type. We observed that ovarian tumor cells isolated from ERα^d/d^ mice express high levels of P450 aromatase protein in luteinized stromal cells of the tumor, suggesting that these cells acquired the ability to synthesize E (Figs. S3B). Furthermore, the activated form of ERα, phosphorylated at Ser-118, is abundantly expressed in the OSC, while aromatase is expressed in ovarian stroma of ERα^d/d^ mice as early as 3 months (Fig. S3C). We postulated that the epithelial ERα signaling remains elevated in response to this locally produced E in ERα^d/d^ ovarian tumors, supporting increased ERα-dependent gene expression, abnormal cell proliferation, and tumorigenesis.

**Figure 5:**
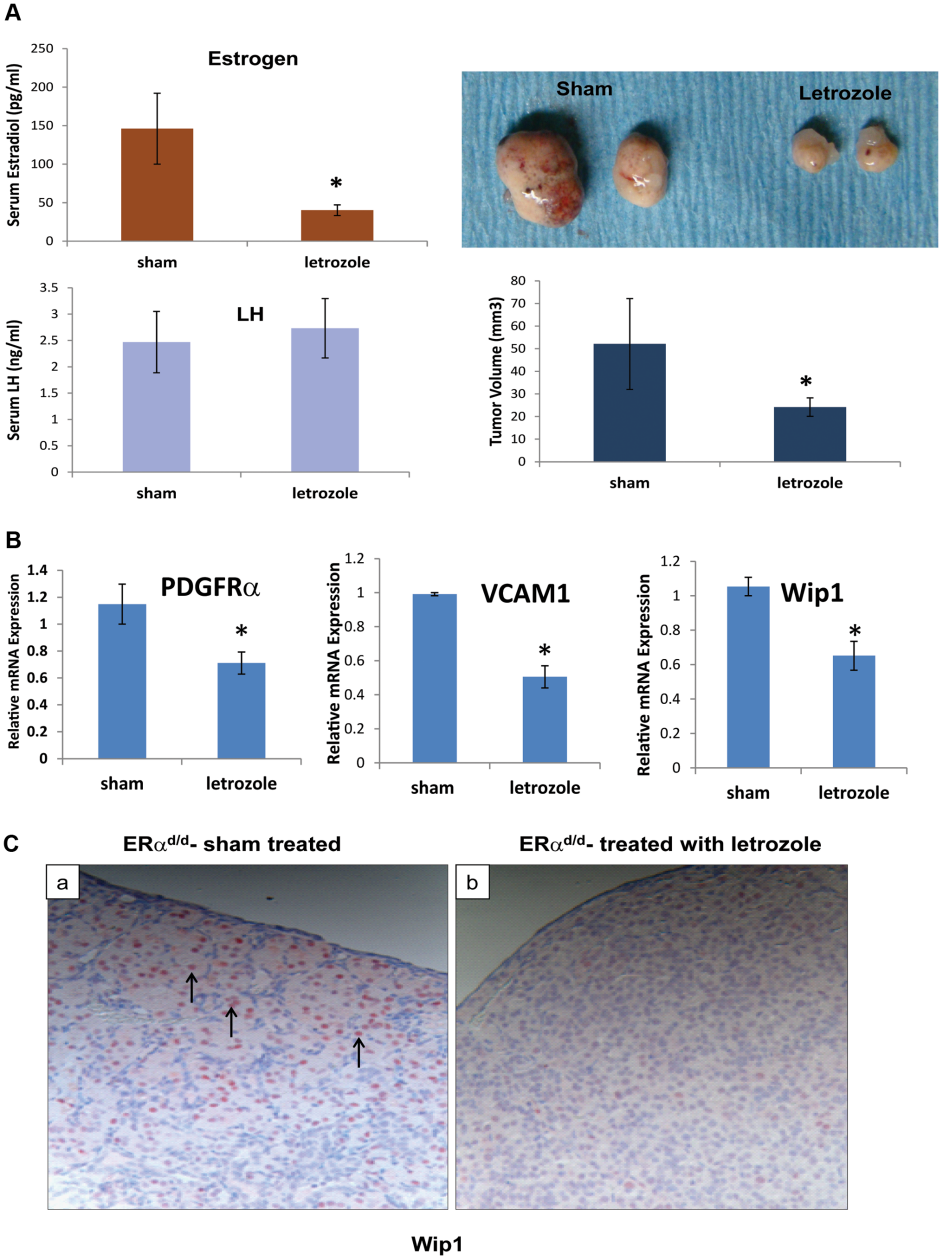
ERα^d/d^ ovarian tumor growth is inhibited by P450 aromatase inhibitor. (**A**) Ovaries with tumors from ERα^d/d^ mice treated with letrozole, a P450 aromatase inhibitor, filled silastic capsules or sham empty silastic capsules for 3 months are analyzed by gross morphology and tumor volume. Serum estradiol and LH is assayed by radioimmunoassay. (**B**) Real-time quantitative PCR was employed to measure mRNA levels of genes associated with ovarian carcinoma, PDGFRα, VCAM, and Wip1, in ovaries with tumors of ERα^d/d^ mice treated with letrozole-containing or sham empty silastic capsules. (**C**) Wip1 protein localization in ovaries with tumors of ERα^d/d^ mice either treated with sham control (a) or treated with letrozole (b). Arrow points to nuclear expression of Wip1. * indicates p<.05. Each treatment group had n = 8 samples.

To examine whether E plays a critical role in ovarian tumor progression in ERα^d/d^ mice, we chronically treated these mice at 3 months of age with letrozole, a specific inhibitor of P450 aromatase, by implanting silastic capsules containing this drug. Following three months of letrozole treatment, ovarian tumors of 6-month old ERα^d/d^ mice displayed a remarkable reduction, up to 60%, in tumor volume when compared to sham-treated ERα^d/d^ mice (Fig. 5A). It is important to note that while ERα^d/d^ mice treated with letrozole exhibited significantly lower levels of serum E compared to sham-treated ERα^d/d^ mice, their serum LH levels were not altered in response to this treatment (Fig. 5A). Interestingly, ERα^d/d^ mice treated with the letrozole exhibited significantly lower levels of ovarian expression of PDGFRα and VCAM transcripts compared to sham-treated ERα^d/d^ mice (Fig. 5B). Similarly, the levels of Wip1 mRNA and protein were markedly decreased in ovarian tumors of ERα^d/d^ mice upon letrozole treatment (Fig. 5, B and C). Taken together, these results confirmed that elevated E signaling in the ovarian tumors of ERα^d/d^ mice leads to dysregulated expression of a subset of genes with known links to ovarian epithelial cancer.

## Discussion

Genetically engineered mouse models are considered to be among the most powerful and promising tools presently available for studying the biology of various forms of cancer and for developing therapeutics. Although the creation of mouse models of ovarian cancer has lagged behind models for many other neoplastic diseases, significant advances have been made in the last decade. Orsulic *et al* have shown that p53-deficient ovarian cells engineered to overexpress multiple oncogenes, *c-myc*, *Kras*, and *Akt*, develop ovarian tumors when injected in mice [42]. Similar mouse models for ovarian epithelial tumors were developed via inactivation of various tumor suppressors, such as *Pten*, *APC*, *p53* and *Rb*, through intrabursal administration of adenoviral vectors [43], [44]. Conditional inactivation of multiple genes, such as *Pten* and *Kras* or *PTEN* and *Dicer*, by expression of Cre recombinase driven by the *Amhr2* promoter also led to ovarian cancer in mice [45], [46]. These mouse models have provided compelling evidence that OSE or FTE can be transformed by altering the expression of a variety of oncogenic factors or tumor suppressors. Some of these models display tumor histotypes similar to ovarian cancer subtypes seen in women. However, it is clear that these models typically require multiple genetic changes and are limited by very rapid tumor onset, which limits their usefulness for studying early modulators of ovarian tumorigenesis. In the present study, we report the development of a unique animal model, in which initiation of ovarian tumorigenesis is independent of any oncogenic insult but dependent on elevated E signaling in the ovary. Since the onset and progression of tumorigenesis is relatively slow in ERα^d/d^ mice, this model is potentially useful in providing insights into the factors involved in the initiation and early phases of ovarian epithelial tumorigenesis.

In ERα^d/d^ mice, ERα is conditionally ablated in the pituitary but retained in the hypothalamus and ovary. The loss of negative-feedback regulation by E in the HPO axis led to elevated production of LH by the pituitary. Interestingly, high levels of gonadotropins in women in early postmenopause have been postulated to play a role in the development of epithelial ovarian neoplasms [4], [5]. Consistent with this notion, it has been found that women with polycystic ovary syndrome, which is accompanied by high LH levels, have a greater risk of developing ovarian cancer [47]. Further supporting a role of gonadotropins in ovarian cancer development, gonadotropin levels in cysts and peritoneal fluid from ovarian cancer patients have been shown to be elevated [48]. However, not all studies have led to similar findings and it is clear that elevated gonadotropin levels alone do not cause ovarian cancer. In fact, our studies using the ERα^d/d^ model suggested that hyperstimulation of ovarian cells by LH results in increased steroidogenesis, leading to high levels of circulating E as well as locally produced E in the ovarian tissue. High levels of testosterone coupled with increased expression of aromatase in the ovarian tissue would lead to increased synthesis of local E. We propose that this elevated E is an important factor in epithelial ovarian tumorigenesis as it stimulates signaling in the OSE, promoting its proliferation and phenotypic transformation. These results are supported by epidemiological and clinical studies, which indicate that postmenopausal women with elevated gonadotropin levels and receiving E replacement therapy exhibit an increased incidence of ovarian tumors [9]–[13]. Consistent with a role of E in the genesis of ovarian tumors, recent reports point to the clinical use of anti-estrogen drugs in stabilization of ovarian cancers [49], [50].

Although many previous studies indicated that epithelial ovarian cancer arises from OSE, recent studies have revealed that the fimbriae of the fallopian tube is a possible site of origin of this malignancy, particularly high-grade serous carcinoma [51]. The common embryologic precursor of OSE and FTE is the coelomic epithelium, which gives rise to the epithelial linings of the fallopian tube and the ovary [27]. Unlike FTE, OSE retains mesothelial characteristics and is not terminally differentiated. It has been proposed that either OSE terminally differentiates to resemble FTE, or the cancer originates in fallopian tube and then spreads to the ovary. In support of the latter hypothesis, a recent study showed that conditional deletion of *Pten* and *Dicer*, using the Amhr2-Cre, led to tumor development in the fallopian tube, which subsequently metastasized to the ovary [46]. Our studies, on the other hand, appear to indicate that ovarian tumorigenesis in ERα^d/d^ mice is associated with differentiation of OSE to FTE. We observed prominent expression of FTE marker proteins, such as PAX8, WT1, and Ber-EP4, which are not normally expressed in OSE, in the ovaries of ERα^d/d^ mice. Furthermore, removal of oviducts from ERα^d/d^ mice did not prevent the onset of ovarian tumorigenesis, indicating that FTE is not the precursor tissue for tumorigenesis in ERα^d/d^ mice.

Interestingly, we did not observe any intraperitoneal metastatic spread of the ovarian tumor in ERα^d/d^ mice. This could be partly due to the fact that the majority of the mutant mice died by 10 months of age due to the enlarged tumor, making it difficult to follow the progression of tumorigenesis beyond this point. The absence of overt malignancy in our model is not entirely surprising as several recent studies indicate that multiple genetic changes are necessary for metastatic transformation. It is conceivable that additional mutation(s) in tumor suppressor genes, such as p53, is required to drive the tumorigenic pathways in ERα^d/d^ ovaries to rapidly progressing ovarian carcinoma, which will culminate in metastasis. Indeed, recent studies, utilizing genomic sequencing data from human high-grade serous ovarian cancer specimens, have shown that these cancers exhibit genomic instability and harbor genetic mutations in p53, Rb, BRCA1, and/or BRCA2 loci [52]–[55].

The ovarian tumors in ERα^d/d^ mice are composed of cells of both epithelial and stromal origins. These tumors appear to be distinct from the tubular or tubulostromal adenomas, which are reported to occur spontaneously in a number of mutant mouse strains, including the W^X^W^X^ mice [56], [57]. The adenomas, composed of numerous tube-like structures plus abundant large luteinized stromal cells, arise due to a defect in primordial germ cell proliferation and rapid loss of oocytes at birth, resulting in destruction of graafian follicles [58], [59]. They also arise when mice are irradiated and there is a rapid loss of oocytes after radiation exposure [60]. However, these adenomas are not lethal and administration of E prevents rather than promotes their development [61]. Furthermore, in contrast to these mutant mouse strains, the ERα^d/d^ mice exhibit normal number of oocytes at 3 months of age, which then start to decline when ovarian epithelial and stromal cells expand and form the tumor mass at 6 months. Therefore, the initial stages of tumorigenesis in ERα^d/d^ mice are independent of the oocyte loss. Most importantly, the growth of the ovarian tumors exhibited by the ERα^d/d^ mice is inhibited by letrozole, indicating that these tumors, unlike adenomas, are E-dependent. The ovarian tumors in the ERα^d/d^ mice are presumably dependent on pituitary LH production, which help luteinize the stromal cells. However, the local production of E by these tumors and the resulting estrogenic effects on ovarian surface epithelial expansion and transformation appear to be the two key features that distinguish these tumors from the endocrinologically inactive tubular adenomas or tubulostromal adenomas.

Although the ovarian neoplasm in ERα^d/d^ mice did not show signs of overt malignancy, there was nevertheless clear evidence of tumorigenic transformation. Particularly striking is the finding that a large number of genes, associated with human serous ovarian cancer, are also expressed in ERα^d/d^ ovarian tumors. Specifically, these tumors exhibit dysregulated expression of PDGFRα, VCAM, and Wip1, which were previously reported to be involved in human ovarian cancer. PDGFRα, a cell surface tyrosine kinase receptor for members of the platelet-derived growth factor family, is over-expressed in human serous ovarian tumors and is targeted in clinical trials to treat ovarian cancers [32], [33]. VCAM, a vascular cell adhesion molecule, is found in the blood circulation of cancer patients and has recently been proposed as a marker to detect early stages of ovarian cancer [34], [35]. Wip1, a p53-inducible phosphatase and an oncogene, is of particular interest. Under normal conditions, it restores cellular homeostasis following DNA-damage by cooperating with p53 to induce G2/M cell cycle arrest, thereby allowing ample time for repair of the damaged DNA [62]. However, amplification of Wip1 leads to sustained inhibition of DNA damage response and tumor suppressors, and consequently, its overexpression has been implicated in a variety of human malignancies, including ovarian carcinoma [37], [63]. Recent studies have revealed that Wip1 is regulated by ERα [64]. Consistent with this finding, administration of letrozole to ERα^d/d^ mice, which decreased the ovarian tumor size, also markedly reduced the expression of Wip1 along with PDGFRα, and VCAM. These results are consistent with our hypothesis that accentuated E signaling in the ovarian tissue promotes aberrant expression of genes that participate in tumorigenesis.

In summary, we describe a unique mouse model that allows us to identify hormonal effectors, particularly elevated E signaling, which play an important role in the development of ovarian epithelial tumorigenesis. In the future, the ERα^d/d^ model will serve as a valuable tool for exploring the involvement of E-dependent signaling pathways in the onset and progression of this deadly disease.

## Materials and Methods

### Animals

Mice (C57BL/6; Jackson Laboratory) were maintained in the designated animal care facility at the University of Illinois College of Veterinary Medicine according to the institutional guidelines for the care and use of laboratory animals. We crossed mice harboring ‘floxed’ ERα gene (Esr1^tm1.2Mma^), termed ERα^f/f^, with PR-Cre mice expressing Cre recombinase under the control of progesterone receptor promoter (Pgr^tm2(cre)Lyd^) to develop mice of genotype Esr1^tm1.2Mma^/Esr1^tm1.2Mma^ Pgr^tm2(cre)Lyd^/Pgr+, which we termed ERα^d/d^. The PR-Cre knock-in mice expression of cre recombinase in pituitary, uterus, oviduct, mammary gland, and corpora lutea of the ovary have been described previously [18]. It has been used extensively to ablate “floxed” genes in tissues expressing PR [17], [18].

### Immunohistochemistry (IHC)

Paraffin-embedded ovarian tissue sectioned at 4 µm, mounted on slides and subjected to immunohistochemistry as described previously [65]. Sections were incubated at 4°C with polyclonal antibodies against PCNA (Santa Cruz sc-56), cytokeratin 8 (Developmental Studies Hybridoma Bank, TROMA I), ERα (Novacastra Laboratories), p-Akt1/2/3 serine 473 (Santa Cruz SC-33437), AMH (Santa Cruz Biotechnology SC-6886), WT1 (Santa Cruz Biotechnology), PAX8 (Proteintech group 10336-1-AP), calretinin (Invitrogen 18-0291), Ber-EP4 (Dako), aromatase (Abcam ab35604), vimentin (Sigma Aldrich V5255). Biotinylated secondary antibodies were used followed by incubation with horseradish peroxidase-conjugated streptavidin (Invitrogen). Sections were stained in AEC Solution.

### Real-time PCR analysis

Total RNA was isolated from ovaries by standard Trizol-based protocols and converted to cDNA. The cDNA was amplified by real-time PCR to quantify gene expression using gene-specific primers and SYBR Green (Applied Biosystems). As a loading control, the expression level of *RPLP0* (*36B4*), which encodes a ribosomal protein, was determined. For each treatment, the mean threshold cycle (*C_T_*) and standard deviation were calculated from *C_T_* values obtained individually from 3 to 4 replicates of that sample. Each sample was subjected to three independent real-time PCR trials. The fold change was derived from the mean *C_T_* values. Primer sequences recognizing each gene are located in Table S1.

### Measurement of serum hormones

Hormones were measured by radioimmunoassay (RIA) at the Ligand Core facility, University of Virginia, Charlottesville. Statistical significance was determined on SAS program using the Tukey procedure to control for comparison-wise error rate. Significance cutoff value of p<.05 was determined to be statistically significant.

### Isolation of cells from ovaries with tumors

Ovarian tumors were removed from mice and digested with either 6 g/liter dispase (Invitrogen) and 25 g/liter pancreatin (Sigma Aldrich), or 0.5 g/liter collagenase (Sigma Aldrich) in Hank's balanced salt solution (HBSS). After incubation for 1 h at 37°C, the tubes were vortexed for 10–12 s until the supernatant became turbid with dispersed cells. The contents were then passed through an 80-µm gauze filter (Millipore). Cells were re-suspended in Dulbecco's modified Eagle's F12 medium (DMEM-F12; with 100 unit/liter penicillin, 0.1 g/liter streptomycin, 1.25 mg/liter fungizone) containing 10% heat-inactivated fetal calf serum. Cell culture was continued for 48 h after addition of fresh medium.

### Immunocytochemistry

Ovarian tumor cells were fixed with 10% formalin solution for 10 m. Cells were treated with 25% Triton X-100 (Sigma Aldrich) in PBS for 10 m and exposed to a blocking serum for 1 h. Cells were treated with primary antibodies and incubated at 4°C and exposed to cy3 or cy5-conjugated secondary antibodies.

### Silastic capsule implant

Silastic capsules were made by filling silastic laboratory tubing with 0.8 mg of ground Novartis Femara tablets (letrozole) and sealing with medical adhesive silicone type A (Dow Corning). For surgery, mice were first treated with analgesic 1 h prior to surgery and then anesthetized. A small dorsal incision was made just below the neck, and the silastic capsule was inserted underneath the skin. The incision was held together with wound clips until healed. After 3 months of exposure to either empty silastic capsules (sham control) or silastic capsules containing letrozole, mice were euthanized and ovarian tumors were fixed or frozen for analysis.

### Statistical analysis

Statistical analysis was performed by ANOVA or two-tailed student's ttest. Values of *P*<0.05 were considered significant.

## Supporting Information

Figure S1 (**A**) Average size and weight of ERα^f/f^ ovaries and ERα^d/d^ ovaries with tumors at 8–11 months of age (n = 62). * indicates p<.05 using two-tailed t-test to calculate statistical significance. (**B**) Histological analysis of ERα^f/f^ ovary and ERα^d/d^ ovarian tumors. Ovarian sections from ERα^f/f^ mice at 3 months of age (a & b) and ovarian sections from ERα^d/d^ mice at 3 months of age (c & d), 6 months of age (e & f), 11 months of age (g & h) are stained with hemotoxylin and eosin. Panels b, d, f, and h are higher magnified images of a, c, e, and g, respectively. (**C**) Survival curve of ERα^f/f^ and ERα^d/d^ mice indicating probability of survival at different weeks of age. Data analyzed using GraphPad Prism. P<.0001 indicated that the survival curves between ERα^f/f^ and ERα^d/d^ mice are significantly different.
(TIF)

Figure S2 (**A**) Similarity between the genetic profiles of aberrantly regulated genes in ERα^d/d^ ovarian tumors compared to the genetic profiles of aberrantly regulated genes in human serous adenocarcinoma from three independent microarray studies. Venn diagrams indicate similarity of aberrantly regulated genes in ERα^d/d^ ovarian tumors compared to aberrantly regulated genes of human serous adenocarcinoma as assessed by microarray analysis published by Adib et al. (A), Hendrix et al. (B) and Lu et al. (C). (D) Table indicates the percentage of aberrantly regulated genes similar between ERα^d/d^ ovarian tumors and human serous adenocarcinoma. Microarray lists indicating aberrantly regulated genes between human serous adenocarcinoma and normal human ovaries were exported from the Oncomine public database. Lists of aberrantly regulated genes between human serous adenocarcinoma and ERα^d/d^ ovarian tumors were compared using Ingenuity software. (**B**) Real-time quantitative PCR was employed to measure mRNA levels of genes associated with ovarian carcinoma, PDGFRα, VCAM, clusterin, and ICAM-1, in ERα^f/f^ and ERα^d/d^ ovaries at 3, 6, and 11 months of age. * indicates significant difference of p<.05 using two-tailed t-test for comparison of gene expressions of ERα^f/f^ and ERα^d/d^ ovaries.
(TIF)

Figure S3 Expression of P450 aromatase in ovarian tumor stromal cells of ERα^d/d^ mice. (**A**) Real-time quantitative PCR was employed to measure mRNA levels of P450 aromatase in ERα^f/f^ ovaries and whole ERα^d/d^ ovarian tumors from mice at 11 months of age. (**B**) Localization of aromatase in cultured ERα^d/d^ ovarian tumor cells is assessed by immunocytochemistry. **Upper**; Epithelial cells dually stained with cytokeratin 8 in red (a), P450 aromatase in green (b), and co-localized with blue dapi staining (c). **Lower**; Stromal cells are dually stained with vimentin in red (d), P450 aromatase in green (e) and co-localized with blue dapi staining (f). Yellow indicates co-localization of P450 aromatase and vimentin (f). (**C**) Localization of aromatase (a,b) in ERα^d/d^ ovarian tumors and in ERα^f/f^ ovaries (c). Ovarian section treated with non-immune IgG (d). Localization of pERα in ERα^d/d^ ovarian tumors (e,f) and in ERα^f/f^ ovaries (g,h). Tissues are from mice at 3 months of age. Red color indicates localization of each protein.
(TIF)

Table S1 Sequences of quantitative PCR Primer Sets. Primer sequences were designed to recognize coding regions of specific mRNA.
(TIF)
