# Supplementary figures and images for: Dysregulated Estrogen Receptor Signaling in the Hypothalamic-Pituitary-Ovarian Axis Leads to Ovarian Epithelial Tumorigenesis in Mice
Source: PLoS Genet. 2014 Mar 6;10(3):e1004230. doi: 10.1371/journal.pgen.1004230 (PMC3945209; doi:10.1371/journal.pgen.1004230)

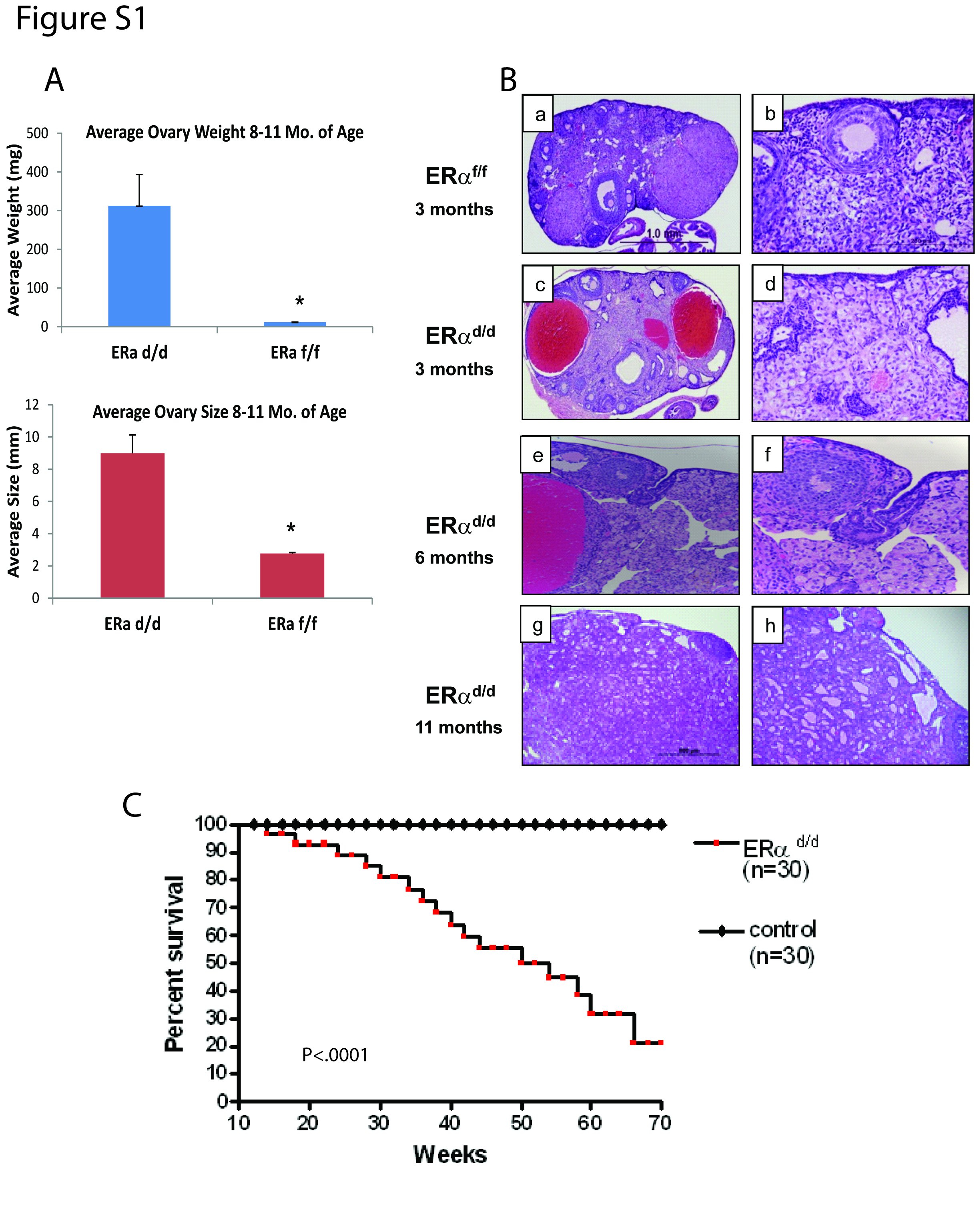

Supplement: Figure S1 — (A) Average size and weight of ERαf/f ovaries and ERαd/d ovaries with tumors at 8–11 months of age (n = 62). * indicates p<.05 using two-tailed t-test to calculate statistical significance. (B) Histological analysis of ERαf/f ovary and ERαd/d ovarian tumors. Ovarian sections from ERαf/f mice at 3 months of age (a & b) and ovarian sections from ERαd/d mice at 3 months of age (c & d), 6 months of age (e & f), 11 months of age (g & h) are stained with hemotoxylin and eosin. Panels b, d, f, and h are higher magnified images of a, c, e, and g, respectively. (C) Survival curve of ERαf/f and ERαd/d mice indicating probability of survival at different weeks of age. Data analyzed using GraphPad Prism. P<.0001 indicated that the survival curves between ERαf/f and ERαd/d mice are significantly different. (TIF) [file pgen.1004230.s001.tif]

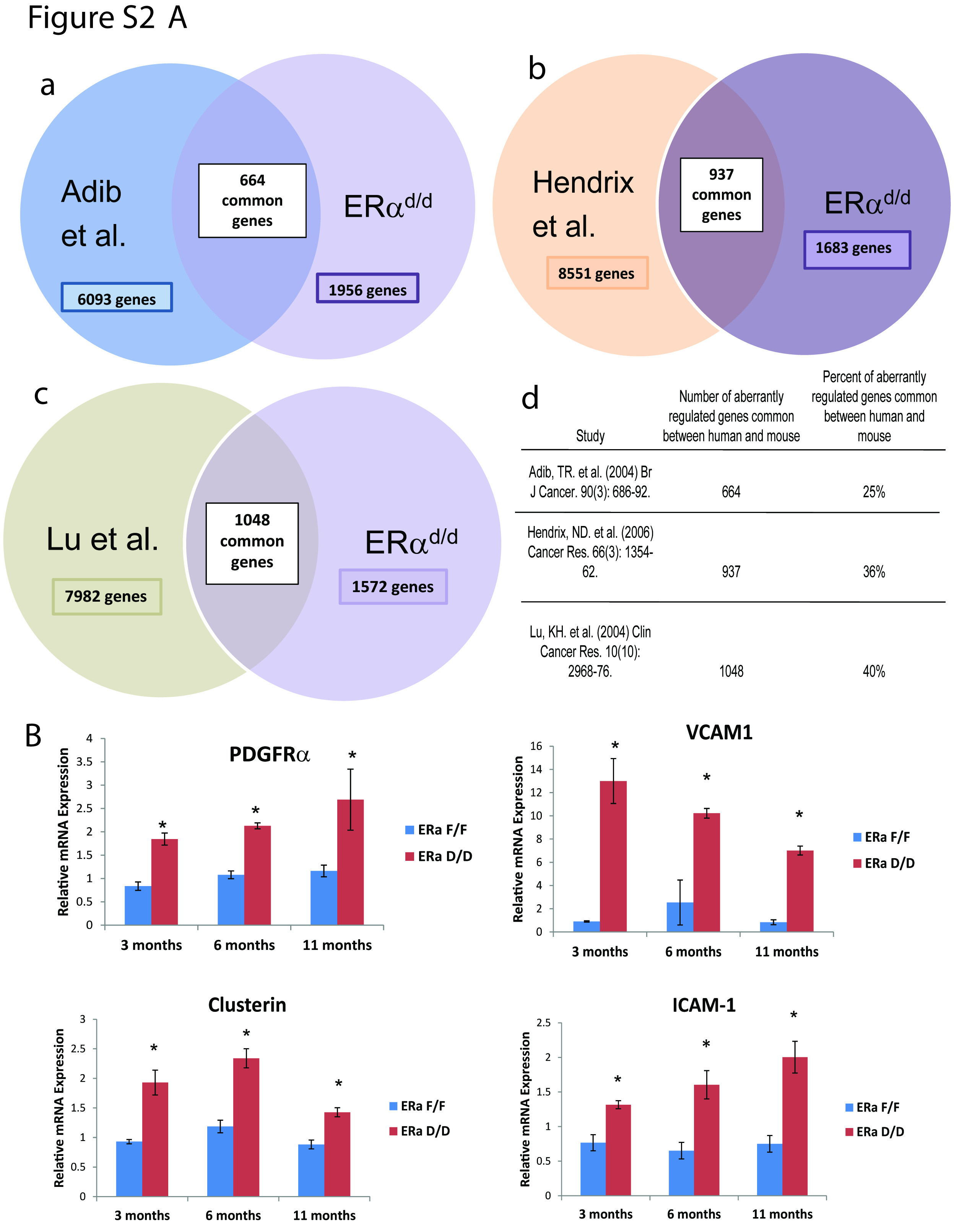

Supplement: Figure S2 — (A) Similarity between the genetic profiles of aberrantly regulated genes in ERαd/d ovarian tumors compared to the genetic profiles of aberrantly regulated genes in human serous adenocarcinoma from three independent microarray studies. Venn diagrams indicate similarity of aberrantly regulated genes in ERαd/d ovarian tumors compared to aberrantly regulated genes of human serous adenocarcinoma as assessed by microarray analysis published by Adib et al. (A), Hendrix et al. (B) and Lu et al. (C). (D) Table indicates the percentage of aberrantly regulated genes similar between ERαd/d ovarian tumors and human serous adenocarcinoma. Microarray lists indicating aberrantly regulated genes between human serous adenocarcinoma and normal human ovaries were exported from the Oncomine public database. Lists of aberrantly regulated genes between human serous adenocarcinoma and ERαd/d ovarian tumors were compared using Ingenuity software. (B) Real-time quantitative PCR was employed to measure mRNA levels of genes associated with ovarian carcinoma, PDGFRα, VCAM, clusterin, and ICAM-1, in ERαf/f and ERαd/d ovaries at 3, 6, and 11 months of age. * indicates significant difference of p<.05 using two-tailed t-test for comparison of gene expressions of ERαf/f and ERαd/d ovaries. (TIF) [file pgen.1004230.s002.tif]

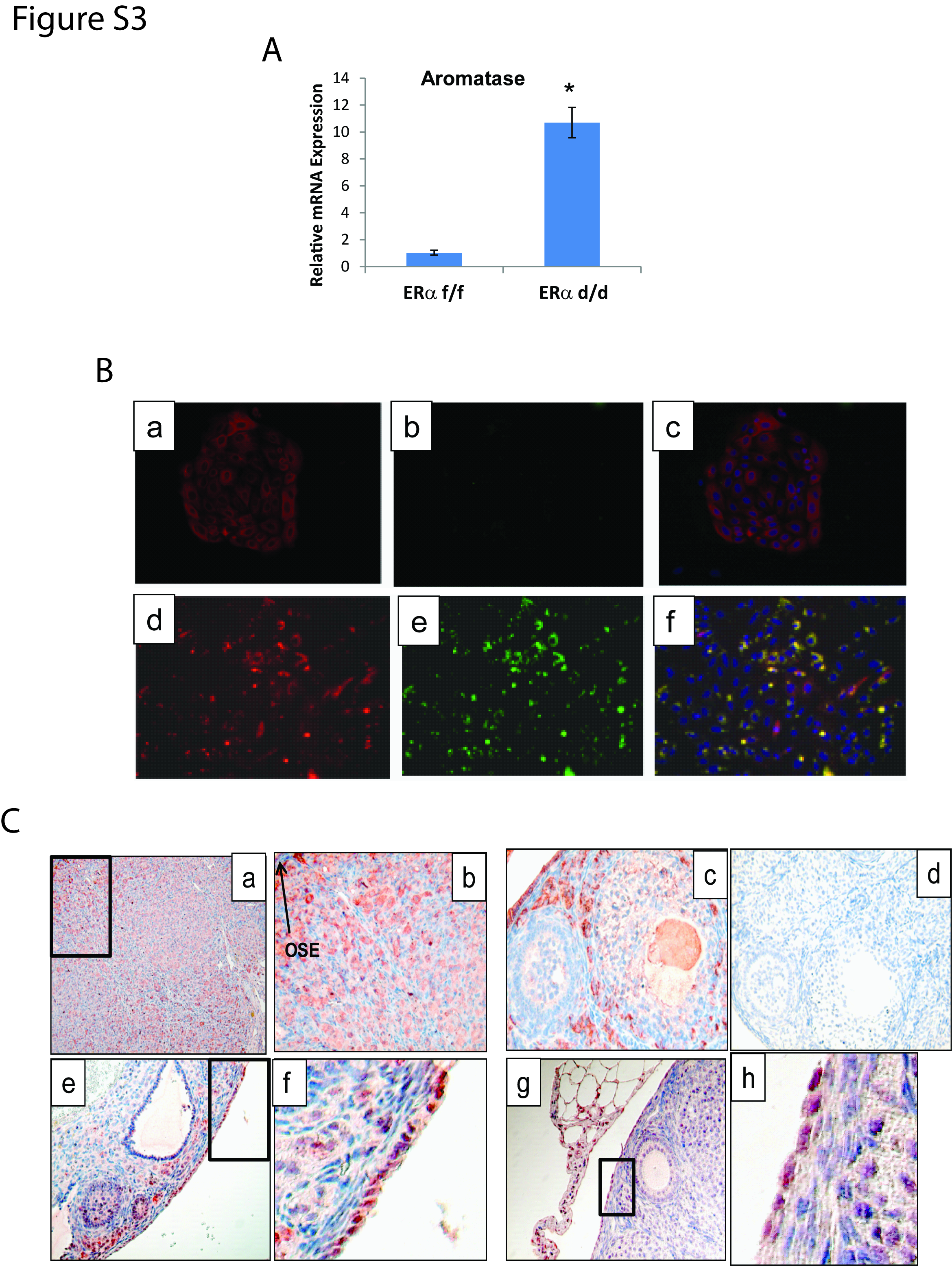

Supplement: Figure S3 — Expression of P450 aromatase in ovarian tumor stromal cells of ERαd/d mice. (A) Real-time quantitative PCR was employed to measure mRNA levels of P450 aromatase in ERαf/f ovaries and whole ERαd/d ovarian tumors from mice at 11 months of age. (B) Localization of aromatase in cultured ERαd/d ovarian tumor cells is assessed by immunocytochemistry. Upper; Epithelial cells dually stained with cytokeratin 8 in red (a), P450 aromatase in green (b), and co-localized with blue dapi staining (c). Lower; Stromal cells are dually stained with vimentin in red (d), P450 aromatase in green (e) and co-localized with blue dapi staining (f). Yellow indicates co-localization of P450 aromatase and vimentin (f). (C) Localization of aromatase (a,b) in ERαd/d ovarian tumors and in ERαf/f ovaries (c). Ovarian section treated with non-immune IgG (d). Localization of pERα in ERαd/d ovarian tumors (e,f) and in ERαf/f ovaries (g,h). Tissues are from mice at 3 months of age. Red color indicates localization of each protein. (TIF) [file pgen.1004230.s003.tif]

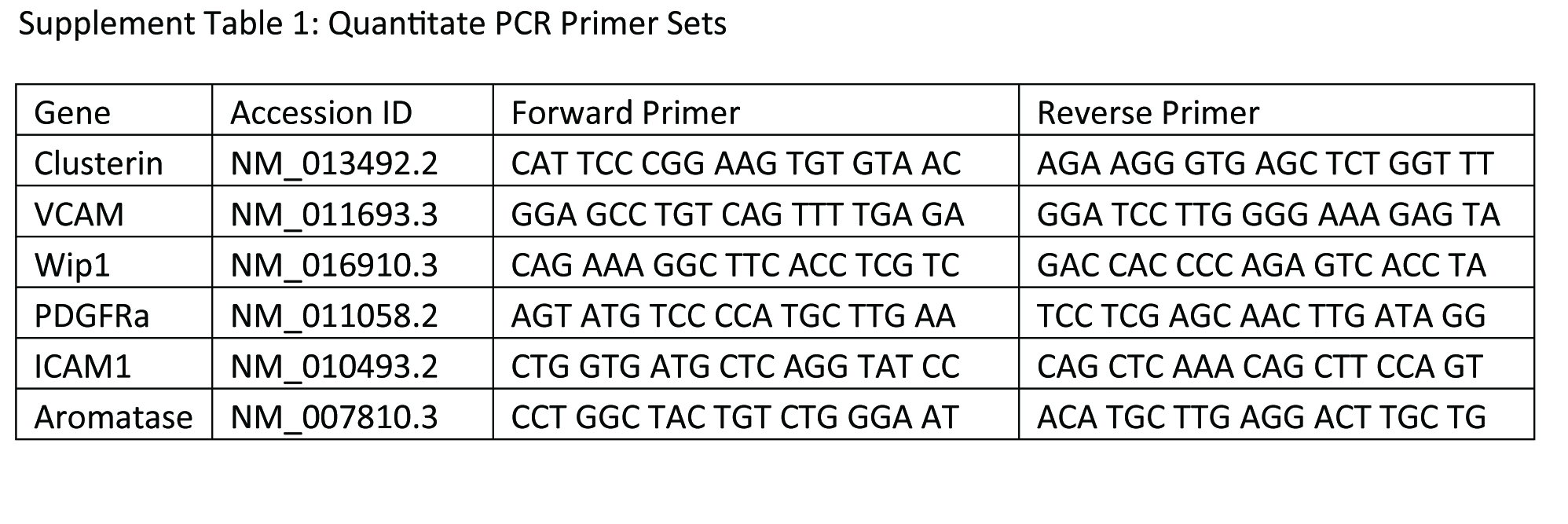

Supplement: Table S1 — Sequences of quantitative PCR Primer Sets. Primer sequences were designed to recognize coding regions of specific mRNA. (TIF) [file pgen.1004230.s004.tif]
